# Adherence to Mediterranean Diet and Ocular Dryness Severity in Sjögren’s Syndrome: A Cross-Sectional Study

**DOI:** 10.3390/medsci13020064

**Published:** 2025-05-23

**Authors:** Celine Chaaya, Elie Raad, Francesca Kahale, Elias Chelala, Nelly Ziade, Georges Maalouly

**Affiliations:** 1 Faculty of Medicine, Hotel Dieu de France Hospital, Saint Joseph University, Beirut 166830, Lebanon; 2 Department of Ophthalmology, Massachusetts Eye and Ear Infirmary, Boston, MA 02114, USA

**Keywords:** Sjogren’s syndrome, ocular dryness, mediterranean diet

## Abstract

**Background**: Sjögren’s syndrome (SS) is a multifaceted clinical condition characterized by various features, including ocular dryness (OD), which plays a substantial role in shaping the clinical presentation of the disease and has detrimental effects on quality of life. Recent research has acknowledged the advantages of the Mediterranean diet (MD) for its positive impact on various autoimmune diseases. This study aims to investigate the correlation between the severity of ocular symptoms in individuals with SS and adherence to the MD. **Methods**: This was a cross-sectional observational study of previously diagnosed SS patients recruited from the histopathological and immunological archives of a university hospital. The data were collected through a telephone questionnaire, including demographic and disease data, the Ocular Surface Disease Index (OSDI) score to evaluate the OD severity, and the Mediterranean Diet Adherence Screener (MEDAS) score to determine adherence to the MD. The primary outcome of the study, the correlation between OSDI and MEDAS scores, was evaluated using Spearman’s correlation coefficient. **Results**: The study included 114 patients, with a mean age of 51 (±13.4) years and a female proportion of 86%. OD was documented in 80.7% of the patients. The median OSDI and MEDAS scores were 23 (IQR 10–40) and 8 (IQR 5–11), respectively. A strong negative correlation was observed between the MEDAS and the OSDI scores (ρ = −0.73, *p* < 0.01). Additionally, there was a significant negative relationship between the richness of diet in fatty acids and the OSDI score (ρ = −0.67, *p* < 0.01). **Conclusions**: The study results suggest an association between lower OD severity in patients with SS and adherence to the MD, particularly the components related to polyunsaturated fatty acids consumption. This approach may serve as a complementary strategy with multiple health benefits, alongside conventional treatment options.

## 1. Introduction

In 1933, Henrik Sjögren, a Swedish ophthalmologist, provided the first description of Sjögren’s syndrome (SS), which primarily affects the exocrine glands [1]. SS stands out as one of the most prevalent autoimmune diseases, displaying a broad systemic phenotype categorized into three distinct types: dry syndrome, systemic symptoms, and systemic manifestations. Its multifaceted origin arises from the intricate interplay between environmental, immunological, and genetic factors [2]. The worldwide prevalence of primary SS is 0.06%, and a notable majority of cases occur in women, with a ratio of 9:1 [3,4].

Ocular dryness (OD) emerges as a primary symptom of SS, marked by disruptions in tear film homeostasis, hyperosmolarity, and inflammation of the ocular surface [5,6]. Assessment of OD often involves employing Shirmer’s test, Tear Break-Up Time test, and the Ocular Surface Disease Index (OSDI) score. These diagnostic tools aim to provide objective measures for quantifying ocular discomfort and evaluating ocular surface health [7,8,9].

The initial approach to treating OD involves primary interventions focused on lubrication with artificial tears and mucus-producing eye drops [10]. However, traditional treatments have often provided limited relief. In fact, clinical improvement is subjective and variable, lacking well-defined therapeutic guidelines. Systemic drug treatments have exhibited no discernible beneficial effects [11,12] and mechanical interventions and surgical treatments are typically reserved for advanced cases [13,14]. Therefore, a real unmet need exists in effective therapy for OD, especially since this symptom has a significant negative impact on the patient’s quality of life [15].

Several studies have demonstrated that adherence to the Mediterranean diet (MD) substantially lowers the risk of developing cardiovascular and inflammatory diseases [16]. In particular, adhering to the MD would be beneficial, especially in terms of cardiovascular health for patients with SS. Indeed, as outlined in the study by Carubbi et al., following an MD is linked to decreased disease activity [17]. This implies that dietary changes, including adherence to the MD, may have an impact on the pathological processes associated with OD [18]. The MD provides notable advantages due to its abundant content of fiber, monounsaturated fats, and polyunsaturated omega-3 (Ω3) fatty acids [19]. A few recent studies have suggested that the MD may also have beneficial effects on ocular surface parameters, though its impact specifically on patients with SS remains largely unexplored. Carubbi et al. examined adherence to the MD in SS patients and found associations with cardiovascular risk profiles and systemic inflammatory markers, but did not directly assess ocular parameters [17]. In contrast, our study focuses specifically on OD severity.

Therefore, the primary objective of this study was to evaluate the association between the severity of OD and adherence to the MD in patients with SS. A secondary aim was to explore whether specific components of the MD, particularly those related to the intake of healthy fats, were linked to variations in ocular symptoms.

## 2. Materials and Methods

### 2.1. Study Population

This was a monocentric cross-sectional observational study. Participants were recruited from a tertiary university hospital, Hôtel-Dieu de France. The population consisted of patients with a previously established diagnosis of SS. Participants were recruited from the histopathological salivary gland archives of the Hôtel-Dieu de France, encompassing biopsies of patients examined from September 2018 to November 2023 with a Chisholm and Mason score of ≥3 (or Focus score ≥ 1). In addition, other patients were selected based on positive immunological results for anti-Sjogren’s Syndrome A (anti-SSA) and/or B (anti-SSB) antibodies conducted within the same timeframe at the same hospital. Eligible medical records were manually reviewed by the principal investigator to confirm diagnosis and ensure consistency with the American College of Rheumatology/European Alliance of Associations for Rheumatology (ACR/EULAR) classification criteria. Final inclusion was confirmed in collaboration with each patient’s treating physician. The minimum sample size was calculated using Sealed Envelope Ltd. (2012) to detect a moderate effect size (Cohen’s d ≈ 0.5) with 80% power and a significance level of 0.05, yielding a required minimum of 30 participants per group. With a total sample of 114, this requirement was met for primary analyses.

### 2.2. Questionnaires

All study participants were contacted by telephone for an interview conducted by a single investigator, who is a trained medical student under the supervision of the principal investigator. Oral informed consent was obtained from all participants using a standardized, IRB-approved template. Participants were aware of the objectives and benefits of the study, and they were provided with the principal investigator’s contact information should they wish to withdraw from the study. Confidentiality was maintained during data collection and analysis. The questionnaire collected basic demographic data (age, sex, country of residence, and marital status) and disease characteristics (disease duration, date since diagnosis). Both the OSDI and Mediterranean Diet Adherence Screener (MEDAS) questionnaires were administered during the structured telephone interview after being validated by the ethics committee.

First, the OSDI score, a reliable instrument, was used to measure the severity of OD [7]. OSDI effectively discriminates between normal ocular surface (0–12), minimal (13–22), moderate (23–32), or severe (33–100) OD. The total score ranges from 0 to 100 [7]. In this study, the OSDI score, validated in Arabic, was used as a continuous variable. Second, the MEDAS, a 14-item questionnaire, was used to assess adherence to the MD model. This nutritional quality index has been validated in Arabic in several countries [20,21]. Participants’ adherence to the MD was categorized using the MEDAS score as follows: low adherence (0–5), moderate adherence (6–9), and high adherence (10–14). These cutoffs are consistent with those used in the original validation study [22]. In this study, the score was used as a continuous variable.

To explore the role of polyunsaturated fatty acids, four items from the MEDAs score, fish consumption, the use of olive oil as the main fat source, daily quantification of olive oil, and consumption of nuts/hazelnuts were analyzed separately. These items, scaled from 0 to 4 according to the number of criteria met, were correlated with the OSDI score.

### 2.3. Statistical Analysis

Data were recorded in an Excel file, then analyzed using SPSS software ver. 26 (IBM Corp, Armonk, NY, USA). Demographic and disease data, OSDI and MEDAS scores were presented descriptively. Demographic and disease characteristics were presented descriptively and compared between patients with minimal OSDI (<13) and those with considerable and high OSDI (≥13) as per the validated cutoffs [7]. Given the non-normal distribution of the primary variables, associations between OSDI and MEDAS scores were assessed using Spearman’s rank correlation coefficient. Group comparisons for non-normally distributed variables were conducted using the Mann–Whitney U test for two groups and the Kruskal–Wallis test for more than two groups. OSDI scores were further analyzed based on MEDAS adherence categories (minimal, moderate, and good [22]), time since diagnosis, dietary intake of fatty acids, and a range of demographic and disease-related factors. Variables significantly associated with OSDI scores in the bivariate analysis (*p* < 0.05) were subsequently included in a multivariable linear regression model to control for potential confounders and identify independent predictors of ocular dryness severity.

### 2.4. Ethics

This study was conducted in accordance with the protocol of Good Clinical Practice and the principles of the Declaration of Helsinki and was approved by the Institutional Board Review (IRB) of Saint Joseph University in Beirut (Tfem/2023/17).

## 3. Results

### 3.1. Demographics

**Figure 1:**
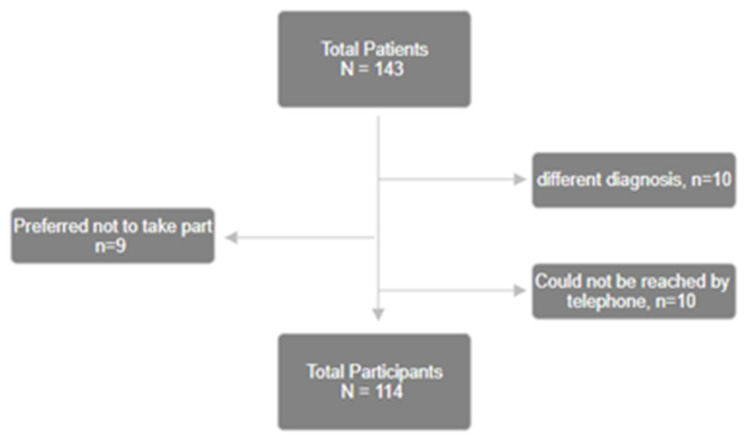
Flow chart of selected participants.

A total of 143 patients were initially contacted. Subjects who did not meet the inclusion criteria (different diagnosis, n = 10) were eliminated from the study. Others preferred not to take part in the study (n = 9) or could not be reached by telephone (n = 10) (Figure 1). Responses obtained from 114 patients were included in the final analysis. The population consisted of 98 women and 16 men, representing a female proportion of 86% (Table 1). The population’s average age was 51 years and ranged from 17 to 86 years, with a standard deviation of 13.4 years. Of note, the age distribution conformed to a normal distribution, as confirmed by the Kolmogorov–Smirnov test. As for the other demographics of this population, 77% of patients were married or in a couple, while 23% were single. Finally, the proportion of smokers in the population was 14%.

### 3.2. Population

The study population consisted of patients with a clinical diagnosis of SS who were monitored by physicians from a variety of specialties (46% rheumatologists, 31% internal medicine doctors, 13% dermatologists). Ninety-three percent of patients reported regular follow-up with their physicians since diagnosis. Out of the 114 subjects, 34 were selected based on positive histological findings (Chisholm and Mason score ≥ 3), while 80 patients were selected based on positive immunological findings (presence of positive antibodies). A total of 80.7% of patients reported the subjective presence of OD. The median OSDI score for the population was 23 (interquartile range (IQR) 10–40), indicating moderate OD (score between 23 and 32). Furthermore, 27.2% of patients had a normal ocular surface (score between 0 and 12), 21.1% had minimal OD (score between 13 and 22), 21.0% had moderate OD and 30.7% severe OD (score between 33 and 100). Adherence to the MD was assessed using the MEDAS score [0–14] as a reference, detecting a median score of 8 (IQR 5–11). Results revealed that 27.2% of patients had minimal adherence to the MD (score between 0 and 5), 33.3% had moderate adherence (score between 6 and 9), and 39.5% of patients displayed good adherence to the MD (score between 10 and 14). Of note, the comparison of the MEDAS score according to marital status (married vs. single subjects) did not identify any statistically significant difference between these two groups (*p* = 0.154).

### 3.3. Correlation Between OD and the MD

**Figure 2:**
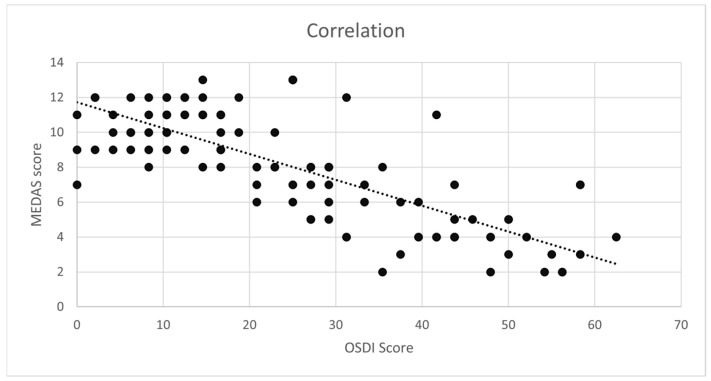
Correlation between the Ocular Surface Disease Index (OSDI) and the Mediterranean Diet Adherence Screener (MEDAS) score in patients with Sjögren’s syndrome. A better adherence to the Mediterranean diet correlated with less ocular dryness.

The MEDAS score exhibited a significant negative correlation with the OSDI score, with Spearman’s coefficient indicating a strong correlation (ρ = −0.73, *p* < 0.01), indicating that higher adherence to the MD was associated with less OD (Figure 2). Furthermore, a significant difference (*p* < 0.01) was observed between the mean OSDI score (13) in patients with good adherence to the MD (MEDAS score ≥ 10) compared to those with poor or moderate adherence to the MD (MEDAS score ˂ 10) with a mean OSDI score of 33 (Table 2). In addition, patients were categorized into two groups based on the time since diagnosis (less than or equal to 5 years and more than 5 years), with 71% of patients being diagnosed within the last 5 years. The correlations between the OSDI and the MEDAS scores were ρ = −0.77 (*p* < 0.01) for those diagnosed ≤5 years ago and ρ = −0.57 (*p* < 0.01) for those diagnosed ˃5 years ago.

### 3.4. Comparison of OD Based on Various Risk Factors

The variation in the median OSDI score according to the different exposure factors was studied. We found no statistically significant association between OSDI and age (*p* = 0.694), sex (*p* = 0.249), smoking (*p* = 0.504), systemic treatment with hydroxychloroquine (*p* = 0.783), ocular treatment with artificial tears (*p* = 0.183) (Table 3). The patients included in the study, who were selected based on positive histological findings, had an average OSDI score of 30. This score was significantly higher than that of patients selected based on immunological results, which was 23 (*p* = 0.024). The correlation between OSDI and MEDAS scores was maintained regardless of the subgroup. In patients whose diagnosis was based on histological findings, Spearman’s coefficient showed a negative relationship (ρ = −0.75, *p* < 0.01), which was also present in those diagnosed based on immunological findings (ρ = −0.73, *p* < 0.01). In the multivariable regression analysis, the multiple correlation coefficient R was equal to 0.78. In addition, the independent variables explained 60.8% of the variability of the dependent variable, which is the OSDI score (R_2_ = 0.608) and the adjusted R square value was 0.577. These variables significantly predict the OSDI score [F (8, 101) = 19.58, *p* < 0.005]. However, none of the coefficients were statistically significant (*p* > 0.05 for all the variables) except for the MEDAS score (*p* < 0.001) and sex (*p* < 0.001).

### 3.5. Impact of Polyunsaturated Fatty Acid Consumption

**Figure 3:**
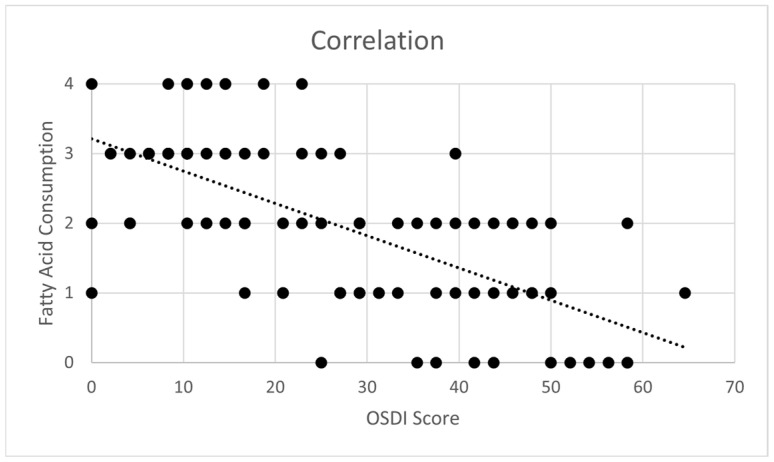
Correlation between the Ocular Surface Disease Index (OSDI) and Fatty acid consumption in patients with Sjögren’s syndrome. A better adherence to fatty acid consumption correlated with less ocular dryness.

There was a significant negative correlation between a diet rich in fatty acids (usage of olive oil as a main culinary fat, consumption of ≥4 tablespoons of olive oil per day, consumption of at least 3 servings of fish or shellfish per week and consumption of at least 3 servings of nuts (including peanuts) per week) and OSDI score, with Spearman’s coefficient ρ = −0.667 (*p* < 0.01) (Figure 3).

## 4. Discussion

This study identified a negative correlation between OD and SS and the adherence to the MD, suggesting a positive effect of the MD on OD, a difficult to treat symptom in patients with SS. The demographic characteristics of our study population reflect the known epidemiology of SS, with a predominance of females (86%) [23]. Moreover, most patients were within the 40- to 60-year age range and were mostly diagnosed within the last five years. These findings agreed with the existing literature on the age of onset of SS, which exhibits a notably high incidence in women during early menopause [23]. Although SS can have a second, earlier peak during adolescence, this group was underrepresented in this sample. This likely reflects real-world referral patterns and the challenges of early diagnosis in younger patients.

The average OD in the population was evaluated as moderate, with a median OSDI score of 23, with most individuals (80.7%) reporting clinical symptoms of OD which confirms its presence in most patients with SS. Adherence to the MD was strongly negatively associated with OD, as demonstrated by the significant correlation between the OSDI and the MEDAS scores, with Spearman’s ρ coefficient of −0.73. This negative correlation implied that as patients adhere more closely to the MD, their OD tends to be less severe, supporting the study hypothesis. These findings suggest that adopting a healthy diet may be associated with a decreased progression of ocular symptoms, or even alleviate them, particularly when adopted promptly following diagnosis. In addition, sex was positively associated with OSDI on multivariable analysis. This is likely due to confounding factors, such as age and comorbidities, which may have masked the association in the unadjusted analysis. However, since this study is cross-sectional study, a causality relationship could not be established, and other longitudinal/interventional studies are needed to determine causality and potentially control for the different cofounding factors that can impact this association.

When diagnosing SS, physicians refer to the criteria established by the ACR. Every patient included in the study exhibited a score of three points or more, determined by histological findings and/or the presence of antibodies, alongside notable subjective OD, a characteristic which is present in most patients. This diagnosis was later confirmed with the treatment by physicians. Nevertheless, the new diagnostic criteria do not rely on subjective elements. Due to resource constraints, the administration of additional objective tests, such as the Schirmer test and saliva quantification, was not feasible. Patients, therefore, met the study’s inclusion criterion of a clinical SS diagnosis with histological and/or immunological support, thus presenting a diagnosis of highly probable SS, according to the 2016 criteria [24].

The patients were selected based on histological studies displayed a statistically significant higher OD compared with those identified through the presence of anti-SSA/SSB antibodies (OSDI score of 30 vs. OSDI score of 23). However, this study did not investigate the reasons behind the selection of one test over the other during the initial diagnosis. The histological involvement of the salivary glands may suggest concurrent infiltration of the lacrimal glands, leading to an elevated OSDI score. Furthermore, even with advanced infiltration, a nutritional therapeutic approach could remain effective, given the inverse relationship observed between MEDAS and OSDI scores in patients diagnosed more than five years ago (ρ = −0.57, *p* < 0.01).

In addition, the patients in this study adhered to diverse treatments and maintained varied lifestyles and dietary habits. The potential interference of these diverse characteristics with ocular symptoms cannot be ruled out. To evaluate the impact of factors likely to influence patients’ perception of ocular symptoms, the mean OSDI score was compared for each assessed item. Factors including age, smoking, hydroxychloroquine treatment, as well as the use of artificial tears, did not seem to exert a significant influence on the OSDI score. Patients who refrained from using artificial tears may initially experience less ocular discomfort, providing justification for their decision. However, many individuals use artificial tears for OD, leading to a substantial expenditure of resources without robust evidence of efficacy, as revealed in this study. These results underscore the notion that adherence to the MD may be beneficial regardless of lifestyle habits and comorbidities.

In the literature, a study revealed a positive correlation between the MEDAS score, particularly fish consumption, and SS activity [17]. The present study affirms this outcome, concentrating specifically on the ocular symptoms of SS rather than the overall disease activity.

Moreover, the recommendation of fatty acid consumption prompts us to investigate its impact on the present research. Originally, justification for the use of supplements in OD stems from the efficacy of polyunsaturated fatty acids for other diseases and chronic conditions, notably inflammatory diseases [25]. Indeed, patients with a diet rich in Ω3 showed a 68% reduction in the incidence of OD [26]. The ability of gamma-linolenic acid and linoleic acid to attenuate ocular surface inflammation in individuals affected by SS was also demonstrated. To further our understanding of the role of polyunsaturated fatty acids, a correlation analysis between the OSDI score and fatty acid elements of the MEDAS was conducted. A significant correlation was found, confirming the hypothesis previously put forward. In addition, mean OSDI was significantly reduced in patients with adequate fatty acid intake. These results present interesting perspectives regarding the possible beneficial role of fatty acid supplementation in individuals with SS.

Overall, the MD, rich in omega-3 fatty acids, antioxidants, and anti-inflammatory compounds, may reduce OD severity through several biological mechanisms. Omega-3 fatty acids, particularly eicosapentaenoic acid (EPA) and docosahexaenoic acid (DHA), are known to modulate inflammation by reducing the expression of pro-inflammatory cytokines, such as IL-1β and TNF-α, which are involved in dry eye disease and OD. By improving the quality of the tear film and enhancing tear production, omega-3s may help mitigate symptoms of dry eye disease. Additionally, recent studies have suggested that dietary interventions, particularly those emphasizing omega-3s, can significantly improve tear break-up time (TBUT) and reduce ocular surface inflammation, which may explain the observed reduction in OD severity in individuals adhering to an MD [27,28]. Our findings complement recent literature that explores non-pharmacologic interventions in SS. Recent studies highlighted the role of dietary patterns and anti-inflammatory foods in modulating autoimmune disease expression [29]. Our study was the first to examine the relationship between MD adherence and ocular surface parameters in SS.

Raising patients’ awareness of the impact of a healthy diet could be beneficial, underlying the central importance of the clinician in communicating and educating patients. The goal is to achieve holistic management of SS, considering its psychological, pathophysiological and symptomatic aspects. In fact, the observed association between adherence to the MD and reduced severity of OD symptoms in patients with SS may have important clinical and public health implications. Given the positive properties of key dietary components discussed above, our findings suggest that modifiable lifestyle factors, such as dietary factors, could become an adjunctive approach in managing SS-related ocular symptoms. Adherence to an MD, a low-cost, accessible, and non-pharmacologic intervention, may represent a practical strategy to help alleviate ocular symptoms and improve patients’ quality of life. These results also have potential relevance for patient education and clinical practice. Integrating dietary guidance into routine care for SS patients could empower individuals with actionable strategies to manage their symptoms alongside conventional therapies. The simplicity and accessibility of dietary interventions make them particularly attractive compared to pharmacologic therapies, which may be costly and less tolerated. The results encourage further investigation into the role of diet in modulating inflammation in SS and the development of educational materials that highlight the role of diet in ocular surface health.

These prospects may pave the way for new studies evaluating the effect of fatty acid supplementation on OD in patients. While this approach is already being explored in the literature, it requires specific attention, particularly in individuals with SS.

The limitations lie in the monocentric aspect of the study. Also, an increase in the number of patients would have strengthened the power of the study and generalizability of the results and improved external validity. Moreover, the uniform quantification of artificial tears treatment across the population was not ensured, potentially introducing bias in the results associated with their use. Furthermore, adherence to MD was self-reported, which might induce a classification and reporting bias and the reliance on self-reported dietary data through the MEDAS questionnaire may be subject to recall bias and social desirability bias. Another limitation of this study is the reliance on the OSDI as the sole measure of ocular discomfort. Although it is a widely used and subjective questionnaire, it is inherently influenced by patient perception and may not capture the full spectrum of OD. Objective clinical tests such as Schirmer’s test and TBUT could provide more accurate and quantifiable measures of tear film function. Finally, a key limitation of this study is its cross-sectional design, which does not allow for assessment of changes over time or control of different covariables; it precludes establishing causality between dietary intake and symptom severity. Future prospective, longitudinal multicenter studies are needed to evaluate over time the causal relationship between adherence to the MD and ocular surface improvement in patients with SS.

## 5. Conclusions

OD represents one of the challenges of the 21st century, impacting a portion of the population, particularly those with SS. This study showed a negative correlation between OD and MD, underscoring the significance of natural approaches in addressing ocular symptoms, especially in the early stages of the disease. A moderate consumption of polyunsaturated fatty acids appears to be beneficial for patients.

It would be relevant to provide recommendations encouraging the adoption of a tailored MD within the population, particularly among individuals with SS. This could potentially contribute to the beneficial management of their symptoms.

## Figures and Tables

**Table 1:** Distribution of patient’s and disease’s characteristics.

	All	Ocular Surface Disease Index		*p*-Value
		Minimal (<12)	Considerable or High (>13)	
N	114	31	83	
Age, mean (SD)	51 ± 13.4	49.0	52.2	0.293
Sex, N (%)				0.042
Female	98 (86)	30 (26)	68 (60)	
Male	16 (14)	1 (1)	15 (13)	
Marital status, N (%)				0.484
Married or in a couple	88 (77)	22 (19)	66 (58)	
Single	26 (23)	10 (9)	16 (14)	
Smokers, N (%)	16 (14)	5 (4)	11 ((1)	0.646
Disease duration of less than 5 years, N (%)	82 (72)	47 (41)	35 (31)	0.323
Diagnosis, N (%)				0.360
Histology (Chisholm and Mason score of ≥3)	33 (29)	7 (6)	26 (23)	
Anti-SSA or SSB antibody	81 (71)	24 (21)	57 (50)	
Treatment with Hydroxychloroquine, N (%)	61 (54)	17 (15)	44 (39)	0.862
Ocular treatment with artificial tears, N (%)	75 (65)	17 (15)	58 (50)	0.134
Associated diseases, N (%)				0.429
No associated diseases	86 (75)	25 (22)	61 (53)	
SLE	13 (11)	4 (3)	9 (8)	
Thyroid	15 (14)	2 (2)	13 (12)	
Presence of subjective OD, N (%)	92 (81)	16 (14)	76 (67)	<0.001
MEDAS, median (IQR)	8 (5–11)	69	45	<0.001
MEDAS score categories, N (%)				<0.001
Minimal	31 (27)	0 (0)	31 (27)	
Moderate	38 (33)	8 (7)	30 (26)	
Good	45 (40)	23 (20)	22 (20)	

Results are presented as frequency, (percentage).

**Table 2:** Comparison of the Ocular Surface Disease Index (OSDI) score based on mediterranean diet (MD) adherence.

	MEDAS	Frequency	Mean ± SD	*p*-Value
OSDI	Poor or moderate adherence	69	33 ± 16.0	<0.01
	High adherence	45	13 ± 8.1	

**Table 3:** Results of the bivariate and multivariable analysis.

	Bivariate Analysis		Multivariable Analysis	
	Correlation with OSDI	*p*-Value	Coefficient	95% CI
Age	0.037	0.694	−0.003	−0.336; 0.329
Sex	0.108	0.249	0.772	0.270; 1.173
Marital status	0.123	0.156	−0.087	−0.472; 0.298
Smoking	−0.063	0.504	−0.037	−0.469; 0.384
Diagnostic modality	−0.192	0.056	0.138	−0.392; 0.667
Treatment with Hydroxychloroquine	0.026	0.783	0.025	−0.280; 0.331
Use of artificial tears	0.205	0.183	0.128	−0.176; 0.431
MEDAS score	−0.73	<0.01	−0.293	−0.344; −0.241

## Data Availability

Data is contained within the article.

## Abbreviations

The following abbreviations are used in this manuscript:

MD	Mediterranean Diet
MEDAS	Mediterranean Diet Adherence Screener
OSDI	Ocular Surface Disease Index
OD	Ocular Dryness
SS	Sjögren Syndrome
